# Immune Signature Against *Plasmodium falciparum* Antigens Predicts Clinical Immunity in Distinct Malaria Endemic Communities*[Fn FN2]

**DOI:** 10.1074/mcp.RA118.001256

**Published:** 2019-10-28

**Authors:** Carla Proietti, Lutz Krause, Angela Trieu, Daniel Dodoo, Ben Gyan, Kwadwo A. Koram, William O. Rogers, Thomas L. Richie, Peter D. Crompton, Philip L. Felgner, Denise L. Doolan

**Affiliations:** ‡ Centre for Molecular Therapeutics, Australian Institute of Tropical Health and Medicine, James Cook University, Cairns, QLD, Australia; § QIMR Berghofer Medical Research Institute, Brisbane, QLD, Australia; ¶ The University of Queensland Diamantina Institute, Brisbane, QLD, Australia; ‖ Noguchi Memorial Institute for Medical Research, University of Ghana, Ghana; ** Naval Medical Research Center, Silver Spring Maryland; ‡‡ Malaria Infection Biology and Immunity Section, Laboratory of Immunogenetics, National Institute of Allergy and Infectious Diseases, National Institutes of Health, Rockville, Maryland; §§ Department of Medicine, Division of Infectious Diseases, University of California Irvine, Irvine, California

**Keywords:** Biomarker: diagnostic, malaria, immunology, clinical data, modeling, antigen signature, feature selection, machine learning, Plasmodium falciparum, protein microarray

## Abstract

A large body of evidence supports the role of antibodies directed against the *Plasmodium spp.* parasite in the development of naturally acquired immunity to malaria, however an antigen signature capable of predicting protective immunity against *Plasmodium* remains to be identified. Key challenges for the identification of a predictive immune signature include the high dimensionality of data produced by high-throughput technologies and the limitation of standard statistical tests in accounting for synergetic interactions between immune responses to multiple targets. In this study, using samples collected from young children in Ghana at multiple time points during a longitudinal study, we adapted a predictive modeling framework which combines feature selection and machine learning techniques to identify an antigen signature of clinical immunity to malaria. Our results show that an individual's immune status can be accurately predicted by measuring antibody responses to a small defined set of 15 target antigens. We further demonstrate that the identified immune signature is highly versatile and capable of providing precise and accurate estimates of clinical protection from malaria in an independent geographic community. Our findings pave the way for the development of a robust point-of-care test to identify individuals at high risk of disease and which could be applied to monitor the impact of vaccinations and other interventions. This approach could be also translated to biomarker discovery for other infectious diseases.

## Abstract (precis)

We have established a predictive modelling framework to systematically analyze IgG antibody responses against a large panel of *P. falciparum*-specific antigens and identify a predictive signature of naturally acquired immunity to malaria. Our results show that an individual's immune status can be accurately predicted by measuring IgG antibody responses to a parsimonious set of 15 target antigens. The identified immune signature is highly versatile and capable of providing precise and accurate estimates of clinical protection from malaria in demographically distinct populations.

## Graphical Abstract

**Figure:**
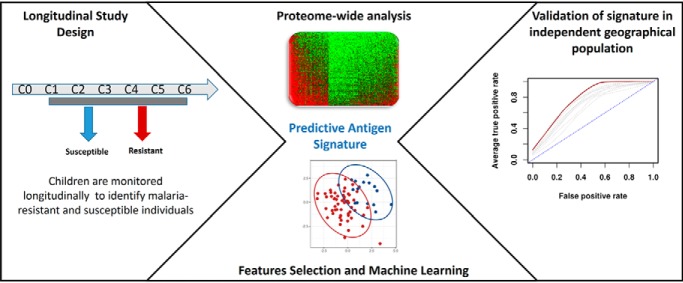

- A predictive modelling framework has been established to analyze IgG antibody responses against a large panel of *P. falciparum*-specific antigens to identify a specific antigen signature of NAI.
- An individual's immune status can be accurately predicted by measuring IgG responses against a small set of 15 defined parasite antigens.
- Proteins identified in the 15-antigen signature represent potential candidates for next-generation malaria vaccines or biomarkers for monitoring the impact of malaria interventions.
- The developed predictive framework can be adapted for developing novel surveillance and intervention tools for other infectious diseases.

Epidemiological and experimental studies support the role of antibodies directed against *P. falciparum* antigens in protective immunity to malaria (1). However, despite decades of intensive efforts, little is known about the parasite antigens that function as targets of naturally acquired immunity (NAI)^1^ and there are no defined correlates of protection. Several studies by us and others have demonstrated that immunity is associated with combinations of reactivity against multiple antigens, rather than the recognition of any single antigen (2–7). Identifying the key antigens targeted by NAI and understanding how NAI develops and is maintained within a population would be immensely beneficial for the development of a diagnostic tool to assess whether individuals or populations are at a high risk of disease and whether this risk changes after the implementation of malaria control measures. Moreover, the identification of an immune signature associated with clinical protection would facilitate the design and development of an effective malaria vaccine comprising the subset of antigens shown to be associated with protection. The small number of antigens under development as vaccine candidates reflects our current limited understanding of immunity against malaria (8).

To address this, we have pioneered studies using protein microarrays expressing the complete or partial proteome of *Plasmodium* parasites to profile the immune response on a proteome-wide scale in individuals naturally exposed to or experimentally infected with malaria (3, 9–12). Those studies have shown that ∼30% of the proteome is reproducibly recognized and that some antigens are serodominant but other are not (12). Proteome-wide studies provide information on immune responses against a large fraction of the parasite proteome, but the overwhelming amount of generated data has been challenging to analyze and interpret with standard statistical approaches and has limited the success in identifying a protective immune signature. For example, the number of variables (proteins) measured in protein microarray experiments usually far outnumbers the sample size, making data analysis challenging and limiting the applicability of standard statistical tests. Traditional statistical approaches that are based on individual antigens are not robust and accurate enough to predict the immune status at an individual level. To overcome these limitations, multivariate methods and machine learning techniques have been recently applied for the analysis of high dimensional “omics” datasets (transcriptomics, metabolomics, proteomics, metagenomics, etc.) to identify predictive biomarker signatures of vaccination, infection or exposure. Insights into the mechanisms of natural and vaccine-induced immunity have been reported for several diseases, including yellow fever, influenza, and tuberculosis (5, 13–16), but not malaria.

Herein, we have established a predictive modeling framework combining feature selection and machine learning to systematically analyze IgG antibody responses against a large panel of *P. falciparum*-specific antigens to identify a predictive signature of clinical immunity to malaria. In a longitudinal study in Ghana, blood samples were collected from well-defined cohorts of young children in the process of acquiring clinical immunity to malaria during multiple time points of the malaria season. Sera were probed against a protein microarray containing 1,080 *P. falciparum* antigens. By analyzing the antibody profiles before the malaria season, we were able to identify a parsimonious set of antibody responses that could predict an individual's immune status (clinically resistant or susceptible) with high accuracy (86%). We further validated this signature in a distinct epidemiological and demographical setting, among 2–10 year-old children and 18–25 year-old adults in Mali. The predictive modeling framework presented here proved to be a powerful approach to identify a sensitive and specific immune signature of NAI to malaria.

## EXPERIMENTAL PROCEDURES

### 

#### 

##### Population and Study Design

Studied children were recruited from the Kassena-Nankana District (KND) of the Upper East region of northern Ghana. In this region malaria transmission occurs throughout the year with two main seasons: a dry season from approximately October to April, and a wet season from approximately May to October (supplemental Fig. S1). The characteristics of the area and study details have been published elsewhere (17–23). Briefly, three hundred children were passively followed up over one calendar year (from May 2004 to May 2005) and were visited seven times (every 2 months). Clinical, hematological and parasitological data were collected at the beginning of the study and during each of the seven visits. A blood sample was obtained by fingerpick (∼0.5–1.0 ml) for thick and thin blood film, for serological analysis and rapid malaria diagnostic test (DiaMed Optimal Rapid Malaria test). Children suffering from uncomplicated malaria were treated with chloroquine and sulfadoxine-pyrimethamine (SP) (Fansidar®) as the first-line drugs according to Ghana Ministry of Health policy at the time. Asexual parasites were counted against 200 white blood cells and converted to parasites/μl assuming a density of 8,000 white blood cells/μl blood. Concurrent parasitemia was defined as any documented parasite density at the time of sample collection. Fever was defined as axillary temperature ≥37.5 °C. Hemoglobin level was measured by Hemocue photometer (Leo Diagnostics, Helsinborg, Sweden). Anemia was defined as hemoglobin level < 9 g/dL. Serum samples from 80 subjects (39 young children aged 1–2 years and 41 old children aged 4–5 years) were randomly selected from this cohort to assay total IgG antibody reactivity against 1080 *P. falciparum* proteins or protein fragments by protein microarray.

##### Ethics Statement

The study protocol for clinical specimens complied with all applicable federal regulations governing the protection of human subjects. The protocol was approved by the Ghana Health Service, the Navrongo Health Research Centre, the Noguchi Memorial Institute for Medical Research, the Naval Medical Research Institutional Review Board, the Office of the Special Assistant for Human Subject Protections at the Naval Bureau of Medicine and Surgery, and the Human Subjects Research Review Board of the Army Surgeon General. All study subjects gave written informed consent. The protein microarray studies were approved by the Queensland Institute of Medical Research Human Research Ethics Committee, and the University of California Irvine Institutional Review Board.

##### Protein Microarray Probing

Total IgG antibody reactivity against *P. falciparum was* assayed using a protein array containing 1080 *P. falciparum* recombinant proteins. Proteins were selected for inclusion based on evidence for blood-stage expression by microarray, proteomics, or expressed sequence tags (ESTs), or predicted to be in the blood-stage secretome (PlasmoDB.org). The array was fabricated as previously described (9). Briefly, coding sequences were PCR-amplified from *P. falciparum* (clone 3D7, (MRA-102, MR4)) genomic DNA and cloned into the PXT7 plasmid with a T7 transcription terminator, and tagged with 5′ polyhistidine (HIS) and 3′ hemagglutinin (HA) epitopes. Recombinant proteins were expressed using *E. coli* cell-free *in vitro* transcription and translation reactions (RTS 100 HY kits from 5 PRIME, Gaithersburg, MD) according to the manufacturer's instructions. Protein arrays were printed as previously described, with each recombinant protein spotted once in each array (3, 9). Once printed, the expression of each recombinant protein printed on the array was verified using anti-polyhistidine (clone His-1, Sigma) and anti-hemagglutinin (clone 3F10, Roche) monoclonal antibodies, as previously described (9). Epstein-Barr Nuclear Antigen 1 (EBNA-1) was included on each microarray chip as a positive control, and an empty T7 vector rapid translation system (RTS) reaction (no DNA) as a negative control. Sera were pre-absorbed against *E. coli* lysate in protein array blocking buffer (1:100 dilution) and then 500 μl of the pre-absorbed plasma was added to each protein array and incubated overnight at 4 °C. Serum antibodies were detected with biotin-conjugated goat anti-human IgG secondary antibody (1:1000 dilution) and visualized with a streptavidin P-3-conjugated antibody (1:200 dilution). Air-dried slides were scanned on an Axon GenePix 4300A array scanner (Molecular Devices, CA) and results read as raw fluorescence signal intensities (SI) were quantified using the Axon GenePix Pro 7 software after correction for spot-specific background. IgG antibody reactivity to the positive control (EBNA-1 fragment) was detected in all individuals, whereas there was no detectable reactivity to the no-DNA (data not shown).

##### Array Data and Statistical Analysis

Protein array data were analyzed primarily in R (http://www.r-project.org) and the GMine data-mining server (6) (http://cgenome.net/gmine). Raw data were measured as the mean pixel signal intensity for each spot. After subtracting slide background, the mean intensity of negative-control spots (no DNA) was subtracted from each test spot to adjust for any cross-reaction effects from the *E. coli* vector used to express the proteins. Next, the corrected data were transformed using the variance stabilizing normalization (vsn) method in GMine using the VSN Bioconductor package. Proteins were defined as positive if the transformed signal intensity was larger than the mean plus 2 standard deviations (SDs) of the transformed negative-control spots (no DNA). For each child, the breadth of the antibody responses was calculated as the number of positive responses against the 1080 *P. falciparum* proteins. Antibody profiles against the 1080 *P. falciparum* proteins were associated with multiple explanatory variables using the multivariate statistical methods redundancy analysis (RDA). Age group (young/old children), time of the malaria season (dry/wet), parasite positive (yes/no), hemoglobin level (g/dL), fever (yes/no) and gender (M/F) were included as explanatory variables and the antibody responses matrix was included as dependent variable.

##### Identification of Proteins Associated with Immunity to Malaria

Associations between antibody responses against individual *P. falciparum* proteins at baseline and subsequent protection from symptomatic malaria were identified by logistic regression. Immune status (resistant/susceptible), age and parasite status (parasite positive/parasite negative) at baseline were included as explanatory variables (predictors) and baseline antibody intensity (SI) against individual *P. falciparum* proteins was modeled as dependent (response) variable. The regression model had the form: antibody signal intensity = protection status (resistant/susceptible) + age group (younger/older) + parasite status at baseline (positive/negative). Individuals were categorized as susceptible if they had a recorded episode of symptomatic malaria (clinical disease) within our one-year study period, and resistant otherwise. Symptomatic malaria was defined as febrile illness (axillary temperature ≥37.5 °C) with concurrent *P. falciparum* parasitemia >2500 parasites/μl. To minimize the misclassification bias that arises because of difficulties in ascertaining exposure to the *P. falciparum* parasite (24), we restricted this analysis to 72 children (35 aged 1–2 years and 37 aged 4–5 years) with any documented parasitemia in at least one contact during the longitudinal study. *p* values were Bonferroni corrected to adjust for multiple statistical comparisons and the significance level α was set to 0.05.

##### Identification of a Predictive Immune Signature

To identify predictive immune signatures underlying clinical protection, we employed sparse Partial Least Squares Discriminant Analysis (sPLS-DA) (25). The high dimensionality and sparsity of protein microarray data, where the number of measured variables (proteins) far outnumbers the sample size, represent challenges that affect applicability of standard statistical tests. sPLS-DA is a powerful method for identifying the key variables of complex and sparse omics datasets that are associated with a biological outcome of interest and can be used for feature selection. The sPLS-DA implemented in the MixOmics R package (26) was run via the GMine data-mining server (6). This procedure involves dimension reduction using Partial Least Squares regression (PLS) for discriminant analysis in combination with a Lasso penalization for feature selection. To reduce the dimensionality of the input data for the sPLS-DA to a manageable size, only the subset of *P. falciparum* proteins was included that had a *p* value < 0.1 in the logistic regression (described above) and signal intensity higher in the resistant group. The immune status (susceptibility or resistance) of children during the one-year study period was included as a response variable. Longitudinal changes in antibody responses against individual antigens were identified by mixed-effects linear regression (27), which can account for the repeated measures study design with multiple samples for each subject. Mixed-effects linear regression modeled antibody signal intensity as dependent variable and time point as fixed effect and individual as a random effect. Following sPLS-DA based feature selection, we then employed a Support Vector Machine (SVM) to test the power of the identified immune signature to correctly discriminate between resistant and susceptible individuals. SVMs are a robust and powerful classification technique that have achieved excellent classification abilities for a wide range of applications, but that are not well suited for feature selection. The SVM was trained on the baseline signal intensities of the selected antigens and evaluated by Monte Carlo cross-validation. Iteratively, 30 samples were randomly selected as a training set and the remaining 42 samples were used as test set. The model was then fit to the training data and evaluated on the test set and the predictive accuracy was defined as the number of correct predictions divided by the total number of predictions. This process was repeated 40 times. The predictive performance, of the model was then evaluated by averaging the receiver operating characteristic (ROC) curves across all 40 Monte Carlo cross-validation runs. The ROCR R package was used for plotting ROC curves and determining the Area Under the Curve (AUC).

##### Effect of the Definition of Clinical Malaria on the Predictive Performance of the Signature

We defined symptomatic malaria (clinical disease) as febrile illness (axillary temperature ≥37.5 °C) with concurrent *P. falciparum* parasitemia >2500 parasites/μl. To assess the effect that different clinical case definitions have on the performance of the identified antigen signature in predicting the immune status of an individual, we performed a sensitivity and specificity analysis as described below. We considered a positive episode of symptomatic malaria to be one in which there was both fever and parasitemia greater than a specified threshold. For each parasitemia threshold, we counted the number of individuals who had at least one episode of symptomatic malaria during the study period, and we randomly selected an equal number of resistant individuals from the entire cohort. For each parasite threshold, we trained SVM on the baseline signal intensities of the selected antigens and evaluated the performance by a leave-one-out cross-validation. The process was repeated 5 times for each parasite threshold, each time with a different random subset of resistant children from the cohort as a control group. Each time, sensitivity and specificity were calculated using the following formula:

Sensitivity: TP/(TP+FN)
Specificity: TN/(TN+FP)

Where

TP: true positives (*i.e.* the number of resistant individuals correctly classified as resistant).
FP: false positives (*i.e.* the number of susceptible individuals incorrectly classified as resistant).
TN: true negatives (*i.e.* the number of susceptible individuals correctly classified as susceptible).
FN: false negatives (*i.e.* the number of resistant individuals incorrectly classified as susceptible).

The sensitivity and specificity of the classifier was then averaged across the 5 runs and plotted *versus* the parasite threshold.

##### Validation of the Predictive Immune Signature in an Independent Cohort

The predictive immune antigen signature discovered by sPLS-DA was next validated in an independent cohort of children exposed to malaria. As a validation dataset, we used our previously published protein microarray data generated in a prospective study investigating host immune response to the malaria parasite *P. falciparum* (10) in 225 individuals from Kambila, Mali. In that study, we used a protein microarray consisting of 2320 probes (representing ∼23% of the *P. falciparum* proteome and including all of the 1080 proteins on the array tested herein) to profile host immune response (IgG) against malaria parasite proteins. Plasma samples were collected from each individual before and after the 8-month malaria season. Individuals were classified as resistant (no malaria episode during the 8 months study) or susceptible (≥1 malaria episodes). In the current study, we included the subset of 194 individuals between the ages of 2–10 years and 18–25 years as an independent validation set. Of these, 128 experienced clinical malaria during the follow up (susceptible) and 66 individuals remained asymptomatic (resistant). Signal intensities were transformed and normalized as previously described (6). Using a SVM we assessed if the predictive antigen signature identified in the Ghana cohort was also able to discriminate susceptible and resistant children from the Mali cohort. Using the baseline data of the Mali cohort, a SVM was trained on the signal intensities of our predictive signature discovered in Ghana and evaluated by Monte Carlo cross-validation as described above. Predictive accuracy, sensitivity and specificity were calculated as described above. A modified randomization test was then carried out to evaluate if the achieved classification accuracy was better than expected by chance. In 1000 iterations, a subset of the 1080 *P. falciparum* proteins was randomly selected (a subset with the same size as the predictive antigen signature discovered in Ghana) and a SVM was trained and validated by Monte Carlo cross-validation on this subset. Finally, we counted the number of times that our predictive antigen signature performed better than the 1000 random subsets.

##### Development and Validation of a Simple Diagnostic Decision Rule to Distinguish Resistant from Susceptible Individuals

We aimed to develop a simple decision rule using the 15-antigen signature discovered by sPLS-DA that could be used to adequately distinguish between resistant or susceptible individuals and aid in diagnostic classification. We developed a simple decision rule like the method developed by Pan *et al.* (28).

First, signal intensities of the n-antigen signature were converted to a binary output by assigning 1 (high) to signal intensities above a selected threshold and 0 (low) to signal intensities below the threshold. The level threshold for each antigen was defined as the mean signal intensity of that antigen in the resistant group minus 1 standard deviation for the Ghana dataset and 0.5 standard deviations for the Mali dataset; the cut-offs were different to account for differences in the variance of signal intensities in the two datasets. Next, an “immunity score” was defined as the sum of the binary outputs for the n-antigen signature. Samples with an immunity score greater than *z* (with 1 ≤ *z* ≤ *n*) were classified as resistant and as susceptible otherwise. The optimal z value was determined by calculating predictive accuracy, sensitivity and specificity for all possible *z* in the range 1 to *n*.

##### Conservation of the Antigens in the Predictive Immune Signature

The amino acid sequences from the proteins identified as predictive immune signature were blasted against the entire NCBI dataset for *Plasmodium* (Plasmodium taxid:5820) using protein-protein BLAST, v2.2.29+. P-P BLAST was run without the filter and an E-value cutoff of 1e-15.

The genomes included in the analysis of conservation are all publicly available *Plasmodium falciparum* strains (*n* = 15) and 11 human-infective *Plasmodium* species. Species and strains included are listed as follows: *Plasmodium falciparum 7G8, Plasmodium falciparum CAMP Malaysia, Plasmodium falciparum Dd2, Plasmodium falciparum FCH 4, Plasmodium falciparum HB3, Plasmodium falciparum IGH CR14, Plasmodium falciparum MaliPS096_E11, Plasmodium falciparum NF135 5C10, Plasmodium falciparum NF54, Plasmodium falciparum Palo Alto Uganda, Plasmodium falciparum RAJ116, Plasmodium falciparum Santa Lucia, Plasmodium falciparum Tanzania 200070, Plasmodium falciparum UGT51, Plasmodium falciparum Viet0m Oak Knoll FVO, Plasmodium knowlesi, Plasmodium knowlesi strain H, Plasmodium malariae, Plasmodium ovale, Plasmodium ovale curtisi, Plasmodium ovale wallikeri, Plasmodium vivax, Plasmodium vivax Brazil I, Plasmodium vivax India VII, Plasmodium vivax Mauritania I, Plasmodium vivax North Korean.*

## RESULTS

### 

#### 

##### Characteristics of the Study Population and Clinical Outcomes in Relation to the Transmission Season and Age

A total of 80 children were randomly selected from a previously described longitudinal study in Ghana (23): 39 young children aged 1 to 2 years and 41 children aged 4 to 5 years (supplemental Table S1). *P. falciparum* infection was prevalent during the entire year, with 58–69% of younger children and 69–95% of older children carrying parasites throughout the study period (supplemental Table S1, supplemental Fig. S2*A*). In general, older children showed concurrent parasitemia at the time of sampling more frequently than younger children (supplemental Fig. S2*A*) (*p* = 6.95E-5, Wilcoxon rank-sum test), but with a similar level of parasite density (*p* = 0.934, Wilcoxon rank-sum test) (supplemental Table S1, supplemental Fig. S2*B*). As expected, parasite prevalence and density were significantly higher during the wet season (*p* = 0.032, *p* = 0.0007 respectively; Wilcoxon paired rank-test) (supplemental Table S1). Younger children were more frequently anemic than older children (*p* = 2.51E-7, Wilcoxon rank-sum test), with a higher hemoglobin level during the dry season compared with the wet season (*p* = 4.06E-4, Wilcoxon paired rank-test) (supplemental Table S1).

##### Antibody Reactivity Associated with Age, Gender, Concurrent Parasitemia and Malaria Season

**Fig. 1.**
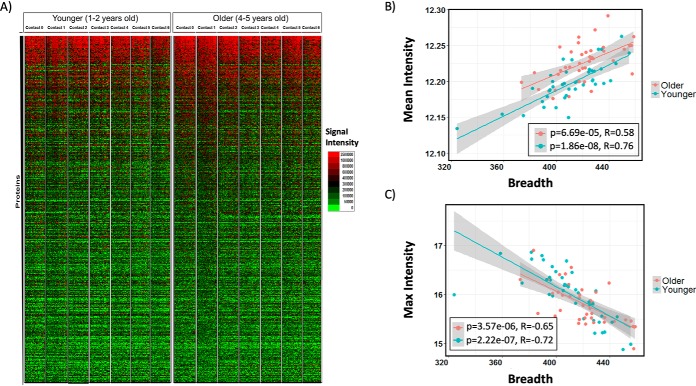
**Longitudinal study of IgG response of 39 young children (1–2 years of age) and 41 older children (4–5 years of age) against *P. falciparum* antigens.** *A*, Heat map of the top 500 most reactive *P. falciparum* antigens by time point ordered by reactivity. Serum samples were collected from each participant every two months, starting at the beginning of the wet season (May 2002) and ending 12 months later at the end of the dry season (May 2003). Antibody responses were profiled using a protein microarray expressing 1080 *P. falciparum* antigens. Antibody responses were generally stronger in older *versus* younger children and for the same children during the wet *versus* dry season. *B–C*, Correlation between mean signal intensity and breadth of response (number of reactive proteins per child) (*B*) and between maximum intensity and breadth of response (*C*). Mean intensities showed a positive correlation with breadth of response whereas maximum intensity was negatively correlated. Breadth of response was defined as the number of reactive proteins per child (at least 2 S.D. above mean of negative controls) and averaged among the six contacts. Mean intensity was calculated as the mean intensities of the 1,080 proteins for each subject and averaged among the 6 contacts. Significance was tested by Pearson's correlation. The red line and the blue line represent the best linear fit for younger and older children respectively. The gray area represents the 95% CI of the best-fit line

Blood samples from each included child (*n* = 80) were collected every two months during the malaria season (7 time points per subject; 539 samples in total) and examined for IgG reactivity against 1,080 individual *P. falciparum* proteins (Fig. 1*A*). As expected, a high degree of variability in antibody responses was observed across individuals, and within individuals longitudinally during the study period. The overall mean of antibody intensity was significantly higher in older children (*p* = 4.85E-05 Wilcoxon rank-sum test), and the mean intensities were higher during the wet season compared with the dry season (*p* = 6.93E-5 Wilcoxon paired rank-test) (supplemental Fig. S3).

Antibody responses to some of the malaria vaccine candidate antigens included in the protein array (erythrocyte binding antigen-175 (EBA175), thrombospondin-related anonymous protein (TRAP), and circumsporozoite protein (CSP)) were generally higher in older children (*p* < 0.05, Wilcoxon rank-sum test) consistent with other studies (2, 10, 21, 29) (supplemental Fig. S4). Conversely, antibody responses against the leading liver-stage vaccine candidates included in the array, LSA-1 and LSA-3, were surprisingly higher in younger children compared with older children (supplemental Fig. S4). Other blood-stage vaccine candidates included in the array showed similar levels of reactivity across the two study groups or higher levels in younger children (*p* > 0.05, Wilcoxon rank-sum test) (supplemental Fig. S4).

Antibody responses showed a gradient of reactivity across the population with some individuals displaying stronger signals and broader recognition compared with others (Fig. 1*A*). Not surprisingly, we observed a positive correlation between the breadth of antibody responses (*i.e.* the number of proteins to which IgG reacted in each subject) and the mean intensity of all IgG reactivity on the array (*p* = 2.826E-13, *r* = 0.70, Pearson's correlation) (Fig. 1*B*). However, unexpectedly, we observed a significant inverse correlation between the breadth of antibody responses and the maximum antibody reactivity (*p* = 3.21E-14, *r* = −0.71, Pearson's correlation) (Fig. 1*C*). The inverse correlation was stronger for younger children (*p* = 2.22e-07, *r* = −0.72, Pearson's correlation) compared with older children (*p* = 3.57e-06, *r* = −0.65, Pearson's correlation). These results suggest that high antibody responses against individual dominant proteins may suppress the response to other protein, resulting in either a low number of highly reactive proteins or a broader number of less reactive antigens. The inverse correlation between breadth and intensity may represent somatic hypermutation and affinity maturation occurring after long term exposure to the parasite. In early years, the antibody responses may be relatively low affinity against a large collection of antigens whereas as the immune response matures over years it may become more focused with higher affinity against a few antigens.

**Fig. 2.**
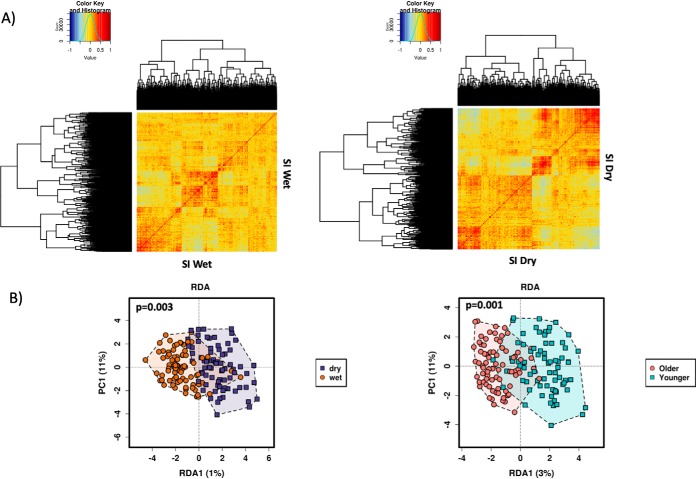
**Antibody responses clustered by age group and time point.** *A*, Pearson's correlation of the antibody responses to the 1,080 *P. falciparum* proteins at a single time point during the wet season (September) (LHS) and dry season (March) (RHS). Antibody responses to the *P. falciparum* proteins were positively correlated in both seasons. *B*, Multivariate statistical methods redundancy analysis (RDA) showed a significant clustering of the antibody profiles against the 1080 *P. falciparum* proteins by time of the malaria season (dry/wet) (*p* = 0.003) (LHS) and by age group (younger/older) (*p* = 0.001) (RHS).

Antibody responses to the 1,080 P. falciparum proteins were positively correlated (Fig. 2*A*), probably as a result of a co-acquisition of antibodies to parasite antigens. However, antibody profiles were highly subject-specific and clustered by age group, and by the malaria season (wet/dry) (Fig. 2*B*). The multivariate statistical method redundancy analysis (RDA) was applied to test the significance of the observed associations. In both the dry and wet seasons antibody responses were significantly associated with age (*p* = 0.001 wet, *p* = 0.002 dry, RDA) and hemoglobin level (hemoglobin level <9 g/dL) (*p* = 0.001 both season, RDA). The presence or absence of fever, gender and parasite density were not associated with antibody reactivity in either of the two seasons (wet season: *p* = 0.500, *p* = 0.704, *p* = 0.526 respectively; dry season: *p* = 0.183, *p* = 0.124, *p* = 0.397 respectively, RDA).

##### Individual Antigens Do Not Predict Protection Against Clinical Malaria

To determine if antibody responses against individual *P. falciparum* proteins could prospectively discriminate resistant from susceptible children, we compared the baseline antibody profiles of children who did not experience any malaria episode during the following one-year study period (*n* = 56) (resistant) compared with those who experienced at least one episode (*n* = 16) (susceptible). Among the 16 susceptible individuals, 13 reported one episode of symptomatic malaria during the year whereas 3 individuals reported two episodes. No significant differences were observed in parasite density (*p* = 0.313, Wilcoxon rank-sum test), breadth (*p* = 0.329, Wilcoxon rank-sum test) or hemoglobin level (*p* = 0.635, Wilcoxon rank-sum test) between resistant and susceptible children at baseline (Table I). In our cohort, none of the 1,080 *P. falciparum* proteins individually was able to discriminate resistant and susceptible children after correcting for multiple testing (Bonferroni *p* > 0.05, logistic regression adjusted for age and parasitemia at the baseline) (supplemental Table S2). Of note, among the antibody responses against the leading malaria vaccine candidates included in the protein array (apical membrane antigen 1 (AMA1), merozoite surface protein 1 (MSP1), merozoite surface protein 2 (MSP2), erythrocyte binding antigen-175 (EBA175)), thrombospondin-related anonymous protein (TRAP), liver-stage antigen 1 and 3 (LSA1-LSA3) and circumsporozoite protein (CSP), none were within the top 100 proteins in discriminating resistant and susceptible children (supplemental Table S2) consistent with recently published findings by us and others (6, 30, 31).

**Table I:** Characteristic of resistant and susceptible children at the baseline (contact 0) Children were defined as “resistant” (*n* = 56) if they did not experience a clinical malaria episode during the 12-month study period and “susceptible” (*n* = 16 children) otherwise. Clinical malaria was defined as fever (temperature above 37.5 °C) and parasite density above 2500 parasite/μl.

Characteristic	Resistant	Susceptible
No. of individuals	56	16
Younger (1–2 years), *n* (%)	26 (46.4)	9 (56.3)
Female sex, *n* (%)	24 (42.9)	8 (50.0)
Parasitemic at time of sample collection, *n* (%)	46 (82.1)	12 (75.0)
Median *P. falciparum* density at time of sample collection (IQR) (parasites/μl)	640 (110–2120)	680 (240–2520)
Median Haemoglobin level (g/dL) at time of sample collection	10.65 (9.56–11.13)	10.50 (9.28–12.03)
Median breadth^a^ at time of sample collection (IQR)	419 (385–437)	424 (407–439)

Only participants who had at least one *P. falciparum* infection recorded in the year were included.

^a^Breadth was defined as the number of positive proteins per individual.

##### Identification of a Predictive Antigen Signature for Protection Against Symptomatic Malaria

**Fig. 3.**
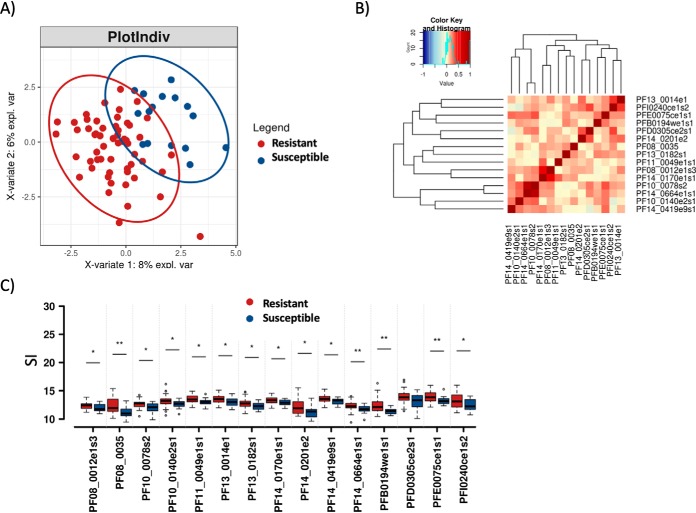
**sPLS-DA identified a 15-antigen signature discriminating susceptible and resistant children.** *A*, sPLS-DA of antibody responses at contact 0 (baseline) showed a separation between resistant (*n* = 56) and susceptible children (*n* = 16). Children aged 1–5 years (*n* = 72) were defined as “resistant” (*n* = 56) if they did not experience a clinical malaria episode during the 12-month study period and “susceptible” (*n* = 16 children) if they experienced clinical malaria. Antibody responses were measured at the beginning of the wet season (contact 0). Ellipses highlight 95% confidence intervals. *B*, Pearson's correlation of the antibody responses against the 15-antigen signature, presented as a heat map. The heat map presents correlations by color code, ranging from blue (negative association) to red (positive correlation). The heat map showed a null or low positive correlation between antigens. *C*, Boxplot of the 15-antigen signature showed a significant difference between resistant and symptomatic children (*p < 0.05, **p < 0.01 *t* test).

Based on our previous observations (3, 6, 9, 10) and other published studies (2, 5–7), susceptibility to symptomatic malaria is more likely to be predicted by measuring antibody responses to a combination of antigens rather than individual antigens. We therefore sought to identify a signature of the most relevant proteins that could predict a child's immune status to malaria. Sparse Partial Least Squares Discriminant Analysis (sPLS-DA) effectively identified a signature of 15 *P. falciparum* antigens capable of discriminating susceptible *versus* resistant children (Fig. 3*A*, supplemental Table S3). As expected, Pearson's correlation of the antibody responses against the 15-antigen signature showed a null or very low positive correlation between antigens (Fig. 3*B*) and a significantly higher intensity in the “resistant” group compared with the “susceptible” group (Fig. 3*C*). Using linear mixed-effects regression, we observed no significant longitudinal changes in antibody reactivity to the antigens in the signature (supplemental Fig. S5), suggesting stability of the antibody signature over different time points of the malaria transmission season.

**Fig. 4.**
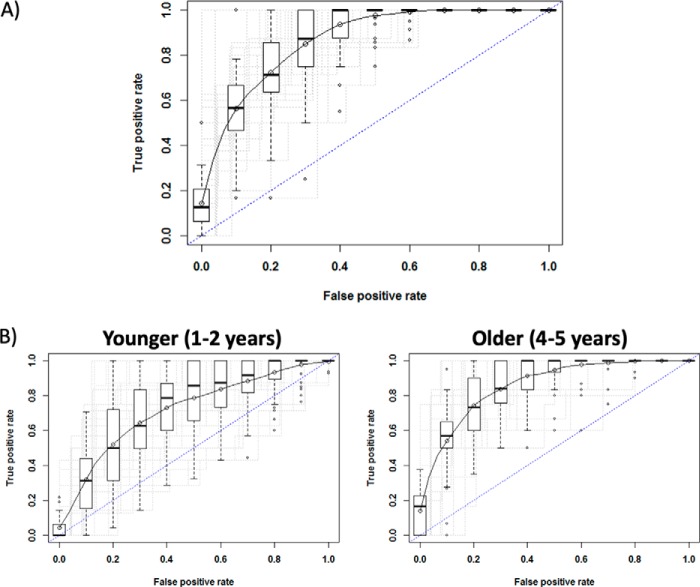
**IgG responses to a signature of 15 selected antigens predicts protection from clinical malaria.** *A,* support vector machine (SVM) was trained on the antibody responses against the 15 *P. falciparum* antigens selected by sPLS-DA to discriminate between resistant and susceptible children. The SVM achieved a classification accuracy of 86%. The performance was evaluated by Monte Carlo cross-validation which randomly selected 30 samples for the training set and 42 for the testing set. The model was fit to the training data, and the predictive accuracy of the model in classifying samples as protected or susceptible was assessed using the testing set. This process was repeated 40 times (dashed black ROC curves). The solid black line represents the mean ROC curve. The dashed blue line represents an AUC = 0.5. *B*, The predictive performance of the SVM was significantly reduced when only young children were included (accuracy 69%) whereas it remained significantly higher when only older children (accuracy 83%) were included.

The discriminatory power of the 15-antigen signature to correctly predict immunity to malaria was examined using a Support Vector Machine (SVM) evaluated by Monte Carlo cross-validation. When all children (1–5 years) were included, the SVM correctly predicted immunity to malaria using the baseline antibody responses against the 15 selected antigens with 86% accuracy, 88% sensitivity, 82% specificity and an AUC of 0.94 (Fig. 4*A*). This predictive performance dropped substantially when the model was applied only to younger children (1–2 years) (accuracy 69%, sensitivity 85%, specificity 85%, AUC = 0.75) but remained significantly high when only older children (4–5 years) were included (accuracy 83%, sensitivity 97%, specificity 88%, AUC = 0.97) (Fig. 4*B*). Because the predictive performance may depend on the clinical case definition used in the study, we considered how an over- or under-estimation of clinical malaria cases might affect the performance of the 15-antigen signature in predicting immunity. As described under Experimental procedures, we considered a series of clinical case definitions of symptomatic malaria. Each definition required the presence of an axillary temperature ≥37.5 °C and a parasitemia above a specified threshold. We modeled the effect of changing parasitemia thresholds on the predictive performance of the 15-antigen signature. Supplemental Fig. S6 shows the sensitivity and specificity of the performance of the 15-antigen signature in predicting symptomatic and resistant individuals as a function of the parasitemia threshold. For resistant individuals, varying the parasitemia threshold across a wide range had a relatively minor effect on sensitivity (supplemental Fig. S6*A*) whereas susceptible individuals were more sensitive to the threshold chosen, varying from 58% to 96% as the threshold varied from any detectable parasitemia to up to 4000 parasites/μl. The sensitivity decreased to 88% at 5000 parasites/μl. The specificity varied greatly for both resistant and susceptible individuals (supplemental Fig. S6*B*), increasing from 64% to 94% and from 71% to 85% from 1 to 4000 parasites/μl respectively, for resistant and susceptible individuals and then decreased to 88 and 79%, respectively, at 5000 parasites/μl.

##### Validation of 15-antigen Signature in an Independent Cohort From Mali

We used previously published protein microarray data from Malian children (10) aged 2–10 years old and adults aged 18–25 years to determine whether the predictive 15-antigen signature identified in Ghanaian children could also predict the immune status in individuals from Mali. Subjects were defined as either resistant if they did not experience a clinical malaria episode during the 8-month study period (*n* = 66), or susceptible if they did (*n* = 128). A SVM trained on the antibody responses at baseline against the 15-antigen signature and evaluated by Monte Carlo cross-validation was able to predict the immune status of Mali individuals with an accuracy of 87%, sensitivity 68%, specificity 82% and an AUC of 0.82. When the model training only included children, the average performance of the model maintained an accuracy 86% and specificity of 73% but with a lower AUC (0.74) and lower sensitivity (33%), as a result of misclassification of resistant individuals.

**Fig. 5.**
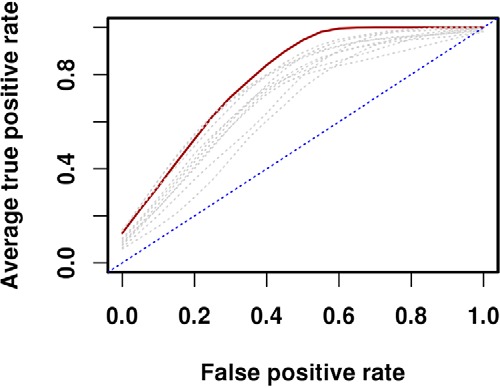
**Validation of the 15-antigen signature in an independent cohort.** The 15-antigen signature discovered by sPLS-DA in Ghanaian children was validated in an independent cohort of 194 children aged 2–10 years old and adults18–25 years old from Mali. First, an SVM was trained on the 15-antigen signature in the Mali cohort and validated by Monte Carlo cross-validation (red ROC curve). Next, 1000 sets of 15 antigens (out of 1080 antigens) were randomly selected and each time trained by SVM in the Mali cohort and validated by Monte Carlo cross-validation. The 15-antigen signature achieved a higher accuracy in 984 out of the 1000 random antigen sets (*p* = 0.016) ROC curves for ten random antigen sets are shown by gray dashed lines. The dashed blue line represents an AUC = 0.5 (*i.e.* random guess of the individual's status).

To assess whether these results were better than those expected by chance, we carried out a re-randomization analysis. From the 1080 *P. falciparum* proteins spotted onto the array, we randomly selected 15 proteins and tested the accuracy in classifying resistant individuals in the Mali dataset by SVM with Monte Carlo cross-validation as described above. The analysis was repeated 1000 times using 1000 random sets of 15 proteins. The 15-antigen signature identified in the Ghana cohort performed better than 984 of the 1000 random sets (*p* = 0.016) (Fig. 5). When the analysis only included children, the number of times the signature performed better than 1000 random sets decreased to 925 (*p* = 0.075), suggesting that the predictive power of the signature increases with the age and/or exposure, as reported in a previous study (2, 30). The ability of the 15-antigen signature to predict the immune status in Mali suggests that the signature may be generalizable across epidemiologically and demographically distinct populations.

##### Development and Validation of a Simple Diagnostic Decision Rule to distinguish Resistant From Susceptible Individuals

**Fig. 6.**
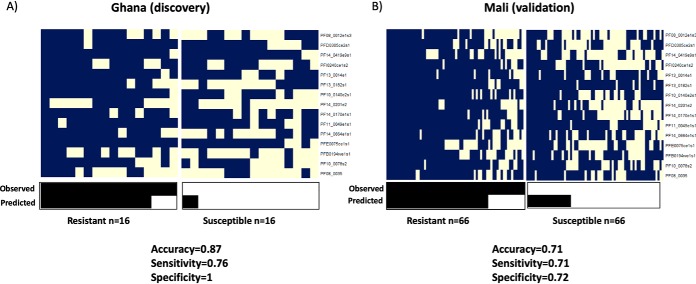
**Performance of the decision rule applied to the 15-antigen signature.** Performance of our decision rule applied to the 15-antigen signature in the discovery cohort of Ghanaian children (*A*) and the validation cohort of individuals from Mali (*B*). Signal intensities were dichotomized using antigen-specific thresholds and binary values of antigens are shown in blue (0/low) and cream (1/high). An individual was then predicted as resistant if at least 11 of the 15 antigens scored high. In the lower heat maps, black and cream represent resistant and susceptible individuals (observed and predicted by the decision rule).

We developed a simple decision rule based on our 15-antigen signature that could be used to distinguish between resistant or susceptible individuals and aid in diagnostic classification. Signal intensities were transformed into binary values (0/1 = low/high) based on defined antigen-specific threshold and individuals were classified as resistant if more than z of the 15 antigens had high intensities (*i.e.* the sum of binary values was greater or equal z). Our 15-antigen signature achieved the best performance for z = 11 with 87% accuracy, 76% sensitivity, and 100% specificity, in our Ghana cohort (Table II; Fig. 6*A*). The performance of the proposed decision rule was evaluated on an independent cohort of individuals from Mali, achieving 72% accuracy, 71% sensitivity and 72% specificity (Fig. 6*B*). The high accuracy observed for the independent validation set demonstrates the robustness of our decision rule in identifying resistant individuals in a different geographical population.

**Table II:** Performance of the decision rule applied to the 15-antigen signature for different z values in the Ghana cohort. For each z value the mean predictive accuracy, sensitivity and specificity were calculated.

z	Accuracy	Sensitivity	Specificity
1	0.5	1	0
2	0.5	1	0
3	0.5	1	0
4	0.5	1	0
5	0.53	0.99	0
6	0.59	0.98	0.14
7	0.65	0.97	0.29
8	0.68	0.95	0.4
9	0.72	0.86	0.51
10	0.80	0.80	0.84
11	0.87	0.76	1
12	0.69	0.45	1
13	0.58	0.29	1
14	0.52	0.06	1
15	0.51	0.03	1

##### Conservation of the 15-antigen Signature

For the purpose of developing vaccines or monitoring tools toward highly variable parasite, the use of conserved antigens across different species and strains is desired. We therefore determined the degree of sequence conservation of our signature among all available *P. falciparum* strains (*n* = 15) as well as the presence and degree of homology for orthologous genes in other human *Plasmodium* species (*vivax, knowlesi, ovale, malariae*) (*n* = 11 strains). Our 15-antigen signature is ∼96% conserved in all *P. falciparum* strains (average percentage of sequence identity across all strains). Nine antigens showed more than 90% of similarity with all (100%) the *P. falciparum* isolated analyzed (supplemental Fig. S7*A*, supplemental Table S3) and 10 antigens shown at least 50% of similarity against the majority of other human infected parasite species (>50%) (*P. vivax, P. malarie, P. knowlesi, P. ovale*) (supplemental Fig. S7*B*, supplemental Table S3). Only one protein (PF3D7_0406100) had a high sequence identity to the human proteome (>50% of identity with more than 50% of coverage) whereas the other 11 proteins showed null or very low degree of homology indicating a low propensity of induction of autoimmune or cross-reactivity (supplemental Table S3).

## DISCUSSION

Advances in “omics” technologies and analytical platforms for complex data have enabled large-scale analyses of natural and vaccine-induced immune responses. Such efforts have revealed novel insights into immunity and correlates of protection. Prominent examples are the immune response to yellow fever vaccination and influenza vaccination (13, 14), and tuberculosis vaccination and infection (15). However, discerning protective immune responses to complex pathogens such as *Plasmodium* spp has proved challenging. Major issues arise from the complexity of the parasite which expressed ∼5300 proteins, the complexity of the host immune response, the high dimensionality of data generated by high-throughput technologies, the often small sample sizes of malaria cohort studies, and the limitations of standard statistical tests in accounting for interactions between different immune responses (30, 32, 33). In this study, we established the utility of a predictive modeling framework, which combines feature selection and machine learning to identify a predictive immune signature of clinical immunity to malaria. We carried out a prospective study of antibody responses against a large panel of *P. falciparum* antigens in a cohort of young children in the process of acquiring clinical immunity to malaria. Children were monitored longitudinally for one year to identify malaria-resistant and susceptible individuals. Using our predictive modeling framework, we showed that an individual's immune status can be accurately predicted by measuring IgG responses against a small set of 15 defined parasite antigens. To determine the reproducibility of this potentially valuable clinical tool for assessing individual malaria risk, we assessed the signature in an independent cohort of Malian individuals between the ages of 2–10 years and 18–25 years. The signature was replicated in this independent cohort, thus supporting the generalizability of our signature to estimate an individual's immune status in different epidemiological settings. Moreover, we showed that all of the antigens included in the 15-antigen signature were relatively stable over the course of the longitudinal study, suggesting that their predictive value would remain stable despite fluctuations in malaria transmission.

This study also highlights the complexity of individual immune profiles. Our results show that an individual's immune profile may reflect either a combination of high antibody reactivity to a low number of antigens, or conversely, low antibody reactivity to a high number of antigens. Therefore, we speculate that high antibody responses against dominant antigens may suppress responses to other antigens. This observation has important implications for vaccine design because it suggests that malaria vaccines based on a single immunodominant antigen or a small number of highly reactive antigens may not be effective in delivering a sufficiently high level of protection. Rather, next-generation malaria vaccines will need to target a combination of many antigens to induce synergistic effects.

The proteins in our 15-antigen signature, identified based on their association with clinical immunity, represent very promising candidates for next-generation malaria vaccines. None of these antigens has yet been studied as a malaria vaccine candidate. Moreover, this specific combination of 15 antigens was a much better predictor of immunity than single antigen, indicating that a vaccine comprising all the 15 antigens identified in our study would most likely induce a synergistic immune response against the parasite. Consistent with the concept that an effective malaria vaccine will likely need to target multiple stages of the *Plasmodium* parasite's lifecycle, all stages of the parasite life cycle are represented by our 15 signature proteins (predominantly pre-erythrocytic stage, 7/15 antigens; and merozoite, 5/15 antigens). These antigens are also conserved among different *P. falciparum* strains and other human *Plasmodium* species making them very suitable for the purpose of developing vaccines or monitoring tools toward highly variable parasite.

One strength of our study is that subjects were regularly contacted every two months, ensuring that most, if not all, clinical malaria episodes were detected and recorded. However, the identification of susceptible children in this age group is problematic as the presence of asymptomatic malaria parasite carriers is common, the clinical signs of malaria are nonspecific, and parasitemia accompanied by a fever may not be sufficient to indicate an episode of clinical malaria (34). We, therefore, investigated the relationship between sensitivity and specificity by varying the parasite threshold in the clinical case definitions of malaria and assessing the impact on the performance of the 15-antigen signature. We found that the 15-antigen signature has an excellent sensitivity and specificity in predicting the immune status of an individual from a threshold of at least 2500 parasites/μl. For parasite thresholds below this cut-off, the sensitivity and specificity decreased as the parasite burden decreased. Because fevers in the presence of low parasitemia may be because of non-malarial causes, it is not surprising that our 15-antigen immune signature would have lower sensitivity and specificity as the parasitemia threshold in the case definition is lowered.

Protein microarrays have proven to be powerful tools for detecting serum antibodies against a vast repertoire of parasite proteins thus offering the opportunity to correlate protective immunity with seroreactivity profiles. Classical univariate approaches to data analysis suggest that a single parasite antigen is probably not enough to elicit (predict) protective immunity, as demonstrated in different epidemiological settings (2–7). These approaches have the disadvantage of considering each variable independently from the others so that the interactions and potential synergies of multi-faceted immune responses are not considered (7, 33). This is an important drawback for the identification of correlates of protection in malaria and the responses to a specific antigen can be associated with protection in some studies or with increasing risk in others (35).

Recent studies have evaluated responses to multiple antigens simultaneously and have suggested that specific antigen combinations may be associated with immunity in field settings (4, 5) and laboratory studies (36). Other studies have shown that a combination of antigens targeting the same stage of the parasite lifecycle does not necessarily produce additive or synergistic protection (37) whereas a more effective combination of antigens may target multiple discrete stages of, for example, invasion (7). These studies represent an important proof-of-concept for the identification of synergetic combinations of antigens, but as they have only included a low number (<100) of antigens, most possible antigen combinations in the *P. falciparum* genome remained unexplored. How to advance beyond those earlier studies has not been obvious. In this study we employed a predictive modeling framework that allowed us to explore antibody responses to 1080 *P. falciparum* proteins. In the computational sciences, machine-learning methods were introduced because of their power to infer associations between correlated features that could not otherwise be made using conventional statistical methodologies (which usually require variable independence) (38–40). Support Vector Machines (SVMs) are now widely applied to transcriptomic, proteomic, and metabolomic datasets to develop predictive models for effective, sensitive and specific decision making. Although SVMs can achieve good predictive accuracy, the excess of features in the training datasets of omics experiments increases both the risk of overfitting and the prediction variability. The selection of a restricted number of variables related to the intended outcome before sample classification eliminates non-informative and correlating variables, thereby increasing the generalization ability of the classifier.

We therefore incorporated a features selection step before building a SVM-based classifier to reduce dimensionality and to make the model less complex and easier to interpret, avoid overfitting and consequently providing a more robust model. Features were selected by sparse partial least squares discriminant analysis (sPLS-DA), which has proven to be particularly appropriate for data with small sample sizes and a large number of correlated variables (25). Although our results show that the signature identified with our approach performs well in an area of intense malaria transmission where clinical disease is generally associated with children below 5 years of age, additional validation in area of lower transmission where many cases occur also in older children and adults is needed.

In summary, we have demonstrated the utility of a predictive modeling framework to decode the host antibody responses to the human malaria parasite *P. falciparum*, in order to identify a specific antigen signature of NAI. In this comprehensive analysis, we have demonstrated that a signature of only a few antigens can be used to predict the immune status at an individual level. Specifically, we have shown that the simultaneous detection of antibody responses to a specific set of 15 antigens is sufficient to discriminate clinically resistant from susceptible children with a very high degree of accuracy and we further validated this in an independent and geographically and demographically distinct cohort of individuals naturally exposed to malaria. We also developed a simple decision rule based on the 15- antigen signature that could be potentially used for seroepidemiological surveillance.

Further evaluation of this signature in multicohort studies would support the utility of this 15-antigen signature as a tool for identifying individuals at high risk of clinical malaria and for monitoring the acquisition and maintenance of NAI. This study provides proof-of-concept for the power of applying advanced analytical approaches such as feature selections and machine learning algorithms to identify a predictive signature of clinical immunity. It paves the way for the development of a robust point-of-care test to identify individuals at high risk of disease and monitor the impact of vaccinations and other malaria interventions.

## Supplementary Material

Supplementary Figures

Supplementary Tables

## Footnotes

^1^ The abbreviations used are:

NAI: naturally acquired immunity
AMA1: apical membrane antigen 1
AUC: area under the curve
CSP: circumsporozoite protein
EBA175: erythrocyte binding antigen-175
EBNA: Epstein-Barr nuclear antigen
LSA1–3: liver stage antigen1–3
MSP1–2: merozoite surface protein 1–2
PCoA: principal coordinates analysis
RDA: redundancy analysis
ROC: receiver operating characteristic
RTS: rapid translation system
SI: signal intensity
sPLS-DA: sparse partial least squares discriminant analysis
SVM: support vector machine
TRA: thrombospondin-related anonymous
vsn: variance stabilizing normalization.
