# Supplementary material for: Immune Signature Against Plasmodium falciparum Antigens Predicts Clinical Immunity in Distinct Malaria Endemic Communities
Source: Mol Cell Proteomics. 2019 Oct 28;19(1):101–13. doi: 10.1074/mcp.RA118.001256 (PMC6944240; doi:10.1074/mcp.RA118.001256)
Supplement: Supplementary Figures [file 142330_1_supp_416764_pzly0k.docx]

**FIGURE S1**: Average seasonal rainfall in the Kassena-Nankana District in Ghana. The wet season is from May (contact 0) to September (contact 2) and the dry season is from November (contact 3) to March (contact 5). Numbered boxes depict months where samples were collected longitudinally.

**FIGURE S2**: Parasite carriage over the longitudinal study. A) Parasite prevalence and B) Parasite density (log 10 transformed) in younger children (1 to 2 years of age) and older children (4 to 5 years of age) at each contact during the study period. Older children were parasitemic more often than young children with a parasite prevalence ranging between 65 to 95% for older children and 58-69% for younger children. No significant difference in parasite density was observed between older children and younger children in any of the contacts (p>0.05 Wilcoxon rank-sum test).

**FIGURE S3:** Mean antibody intensity against the 1,080 *Pf* proteins for younger children (1-2 years old) and older children (4-5 years old) during the longitudinal study: wet season from contact 0 (May) to contact 2 (September) and dry season from contact 3 (November) to contact 5 (March). The values of antibody signal intensity (SI) were averaged for each individual among the 6 contacts or separately for the wet season (contact 0 to contact 2) and for the dry season (contact 3 to contact 5). The difference in mean of the averaged SI between younger and older children were tested by Wilcoxon rank-sum test. Differences in antibody signal intensity between the wet and the dry season were tested for the same individual using Wilcoxon paired rank-test. The mean of the averaged antibody intensity was stronger in older children (p= 4.85E-05 Wilcoxon rank-sum test) and for each individual, the mean intensity in the wet season was stronger than the mean intensity in the dry season (p= 6.93E-5 Wilcoxon paired rank-test)

**FIGURE S4**: Antibody responses against the leading malaria vaccine candidates apical membrane antigen 1 (AMA1), merozoite surface protein 1 (MSP1), merozoite surface protein 1 and 2 (MSP1-MSP2), erythrocyte binding antigen-175 (EBA175)], thrombospondin-related anonymous protein (TRAP), liver-stage antigen 1 and 3 (LSA1-LSA3) and circumsporozoite protein (CSP) in younger and older children along the longitudinal study. The antibody intensities were generally higher in older children for AMA1, EBA175, CSP and TRAP. Higher intensities in younger children were observed for LSA1 and LSA3. No differences between younger and older children were observed for MSP1 and MSP2. Significant differences in antibody intensities between younger and older children in each contact are shown by the red asterisk (Wilcoxon rank-sum test).

**FIGURE S5: Mixed-effects linear regression model for the 15 antigens signature identified from PLS-DA**. Mixed-effects linear regression models included antibody responses against each protein of the signature as dependent variable, contact as fixed effect and individual as a random effect. Out of the 15 proteins of the signature, 14 antigens showed no significant changes in the responses along the study period (p>0.05) while one protein showed only borderline significant variation (p=0.041).

**FIGURE S6:** Effect of the clinical case definition of malaria on the performance of the 15 antigen signature in predicting the immune status of an individual in Ghana. We considered a series of clinical case definitions of symptomatic malaria. Each definition required the presence of an axillary temperature ⩾37.5°C and a parasitemia above a specified threshold. For each parasite threshold we trained SVM on the baseline signal intensities of the 15 selected antigens and evaluated the performance by a leave one out-cross validation. For each parasite threshold, the process was repeated 5 times each time with a different random subset of resistant children from the cohort as a control group. Shown is the averaged sensitivity and specificity (over the 5 runs) of the 15 antigen signature as a function of the parasite threshold used in the clinical case definition.

**FIGURE S7**: Heatmap representing the degree of sequence conservation between the 15 antigens signature amongst all available *P.falciparum strains* (A) and amongst other human infected *Plasmodium* species (B). The percentage sequence identities were obtained by blasting the amino acid sequence of the 15 proteins in the signature against the NCBI dataset for the *Plamodium* genomes (taxid:5820) using protein-protein BLAST v‎2.9.0+ (2019). P-P BLAST was run without the filter and an E-value cutoff of 1e-15. The genomes included in the analysis of conservation are all publically available *Plasmodium falciparum* strains (n=15) and 11 human infected *Plasmodium* species. Species and strains included are listed as follows: *Plasmodium falciparum* 7G8, *Plasmodium falciparum* CAMP Malaysia, *Plasmodium falciparum* Dd2, *Plasmodium falciparum* FCH 4, *Plasmodium falciparum* HB3, *Plasmodium falciparum* IGH CR14, *Plasmodium falciparum* MaliPS096_E11, *Plasmodium falciparum* NF135 5C10, *Plasmodium falciparum* NF54, *Plasmodium falciparum* Palo Alto Uganda, *Plasmodium falciparum* RAJ116, *Plasmodium falciparum* Santa Lucia, *Plasmodium falciparum* Tanzania  200070, *Plasmodium falciparum* UGT51, *Plasmodium falciparum* Viet0m Oak Knoll  FVO, *Plasmodium knowlesi,* *Plasmodium knowlesi* strain H, *Plasmodium malariae*, *Plasmodium ovale,* *Plasmodium ovale* curtisi, *Plasmodium ovale* wallikeri*, Plasmodium vivax*, *Plasmodium vivax* Brazil I, *Plasmodium vivax* India VII, *Plasmodium vivax* Mauritania I, *Plasmodium vivax* North Korean. The colorization scale for the percentage of identity of the BLAST hits is displayed with blue indicating little to no similarity, and red a high degree of similarity.

**FIGURE S1**


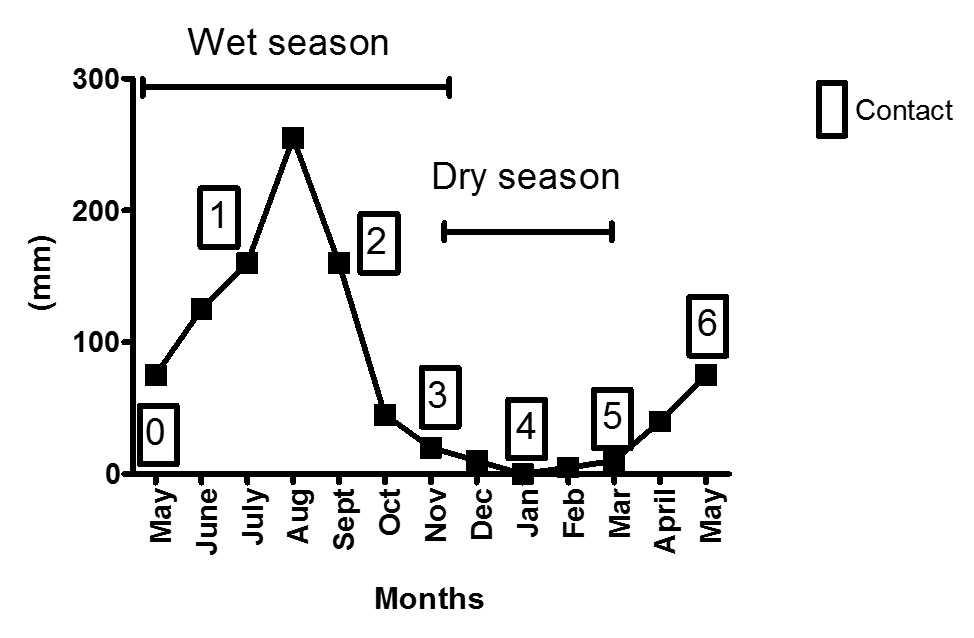


**FIGURE S2:**


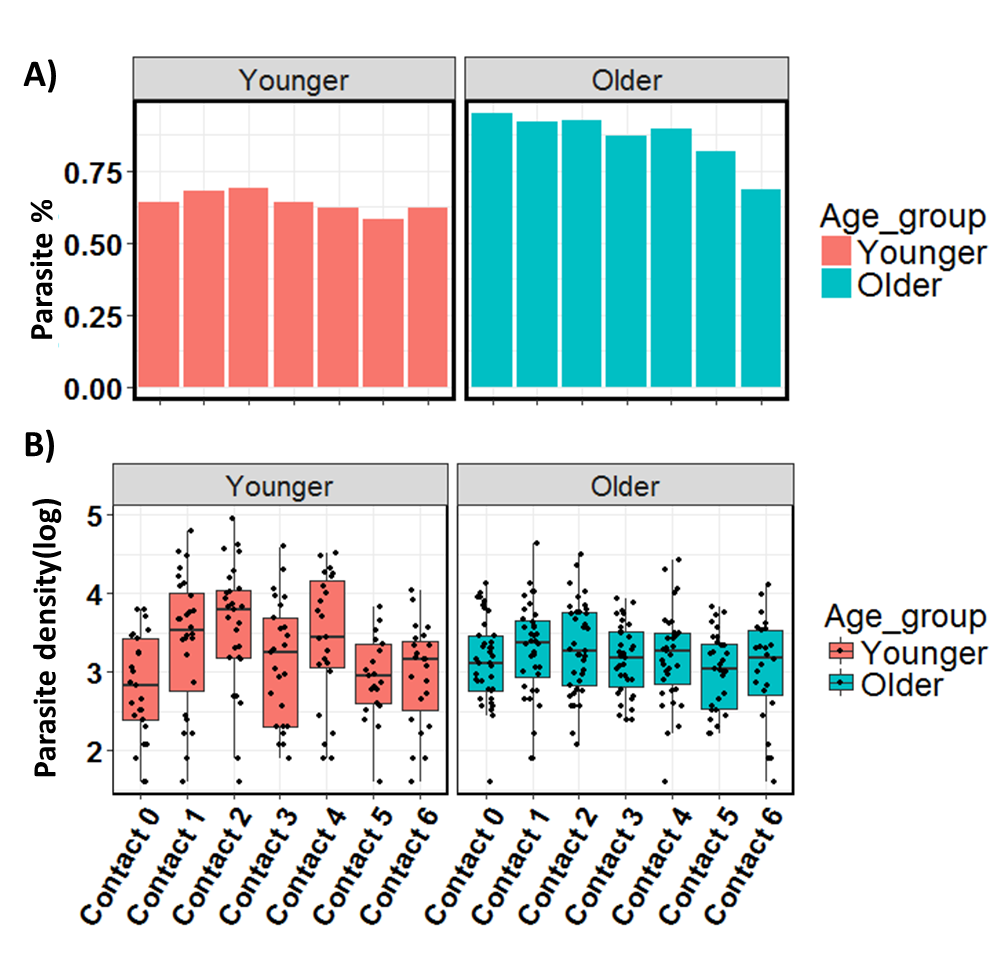


**FIGURE S3**


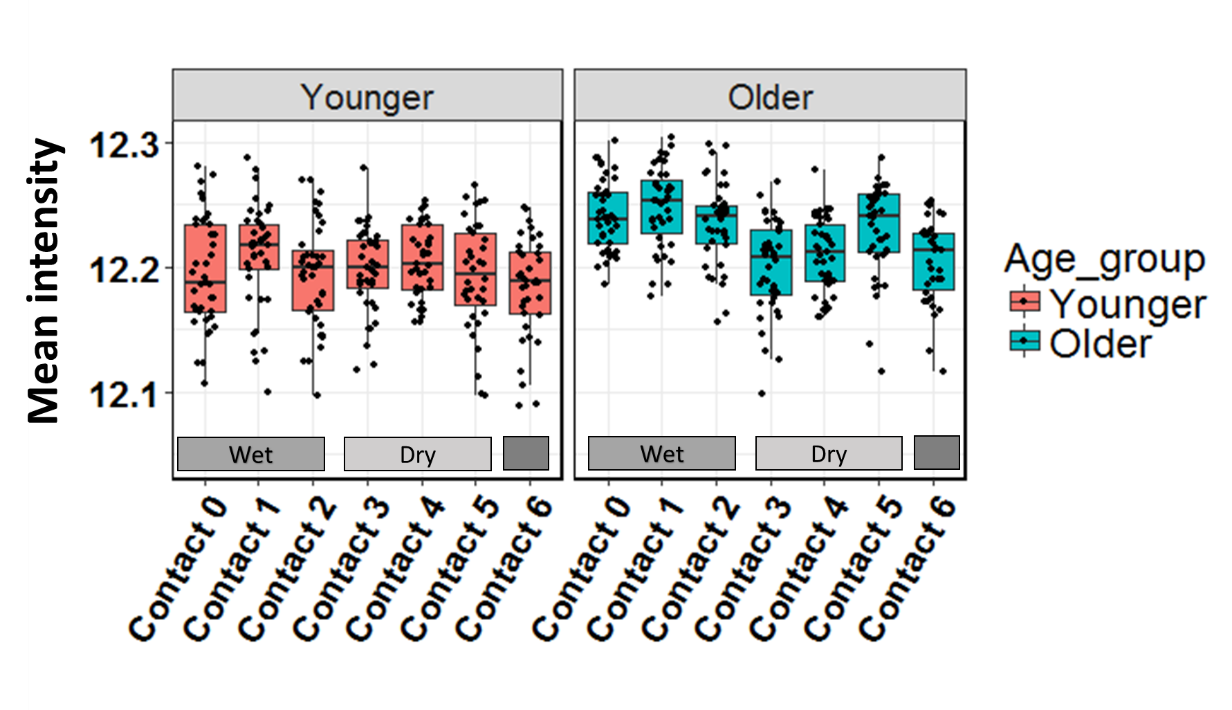


**FIGURE S4**


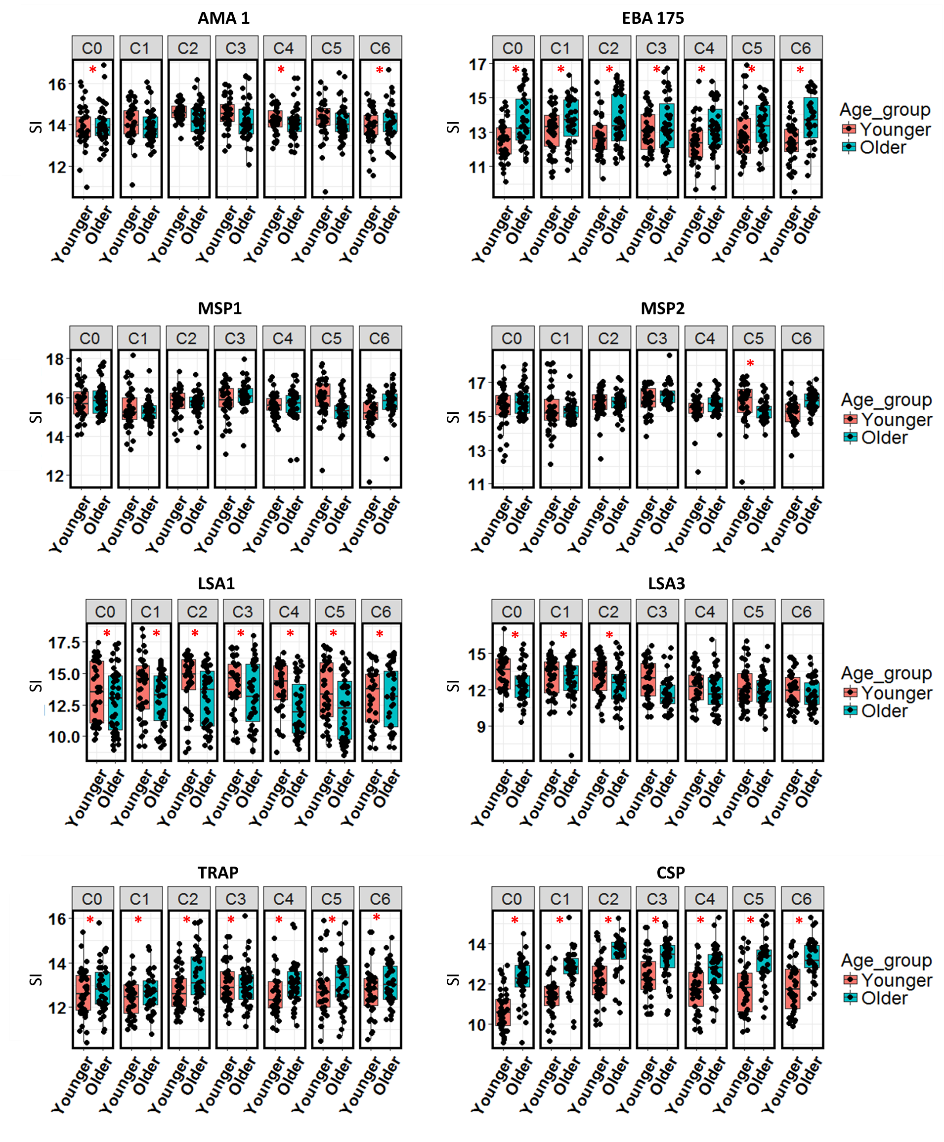


**FIGURE S5**


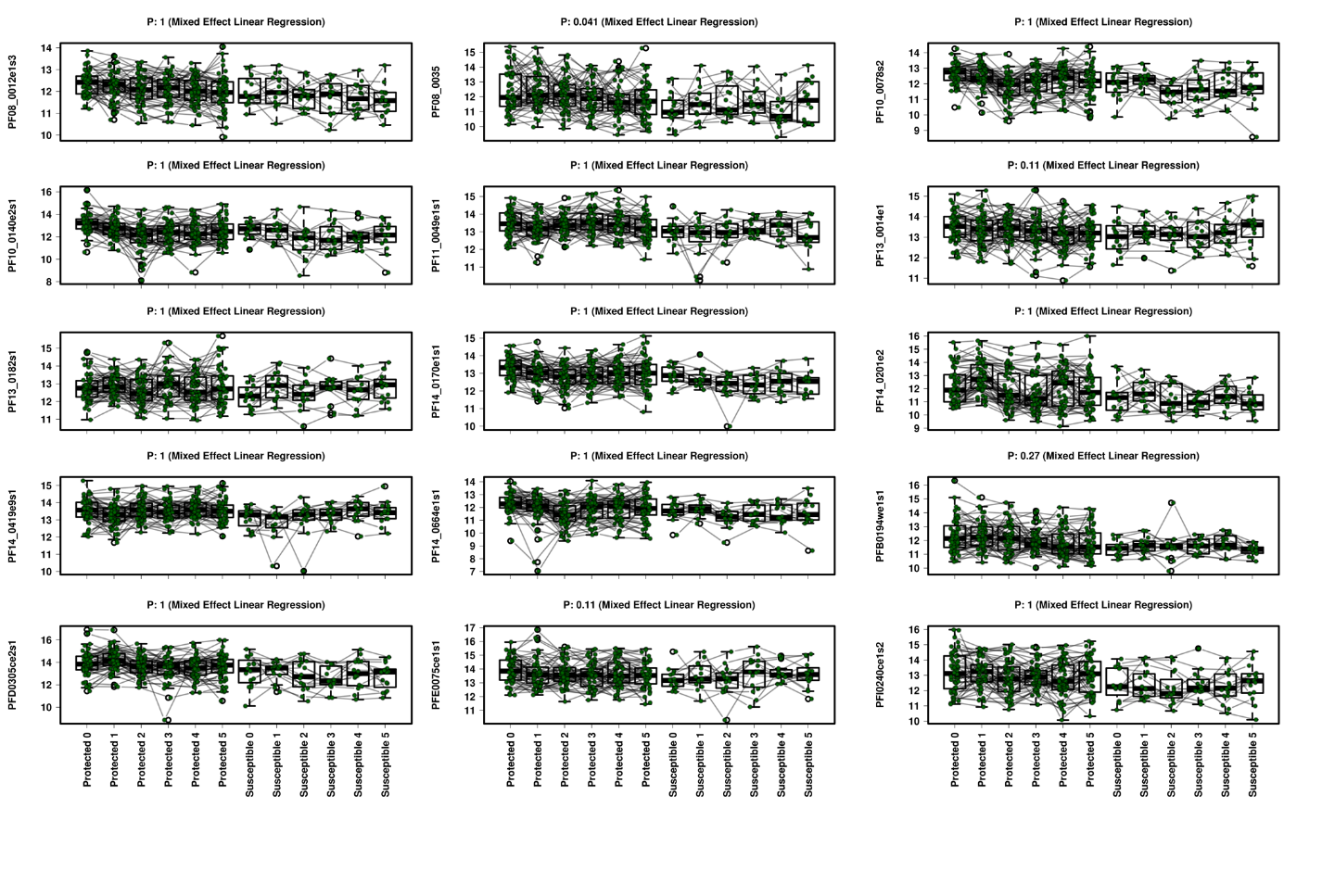


**FIGURE S6:**

**
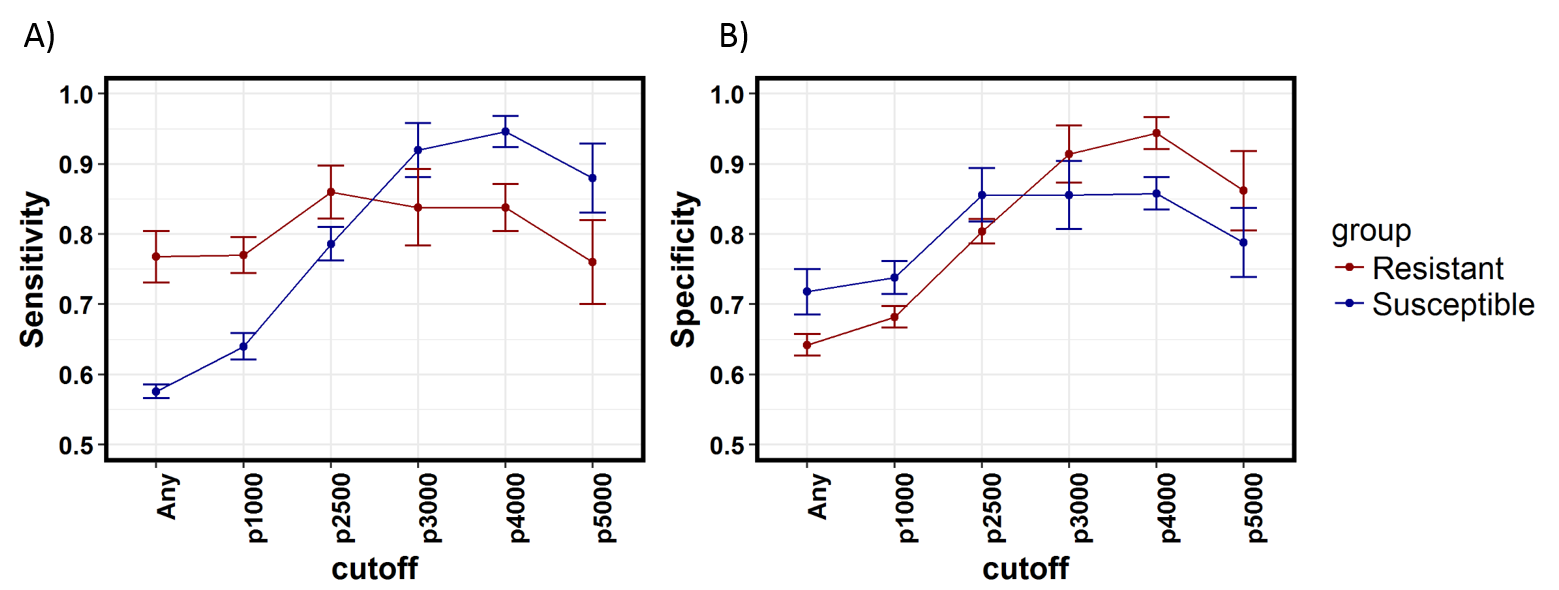
**

**FIGURE S7**

**
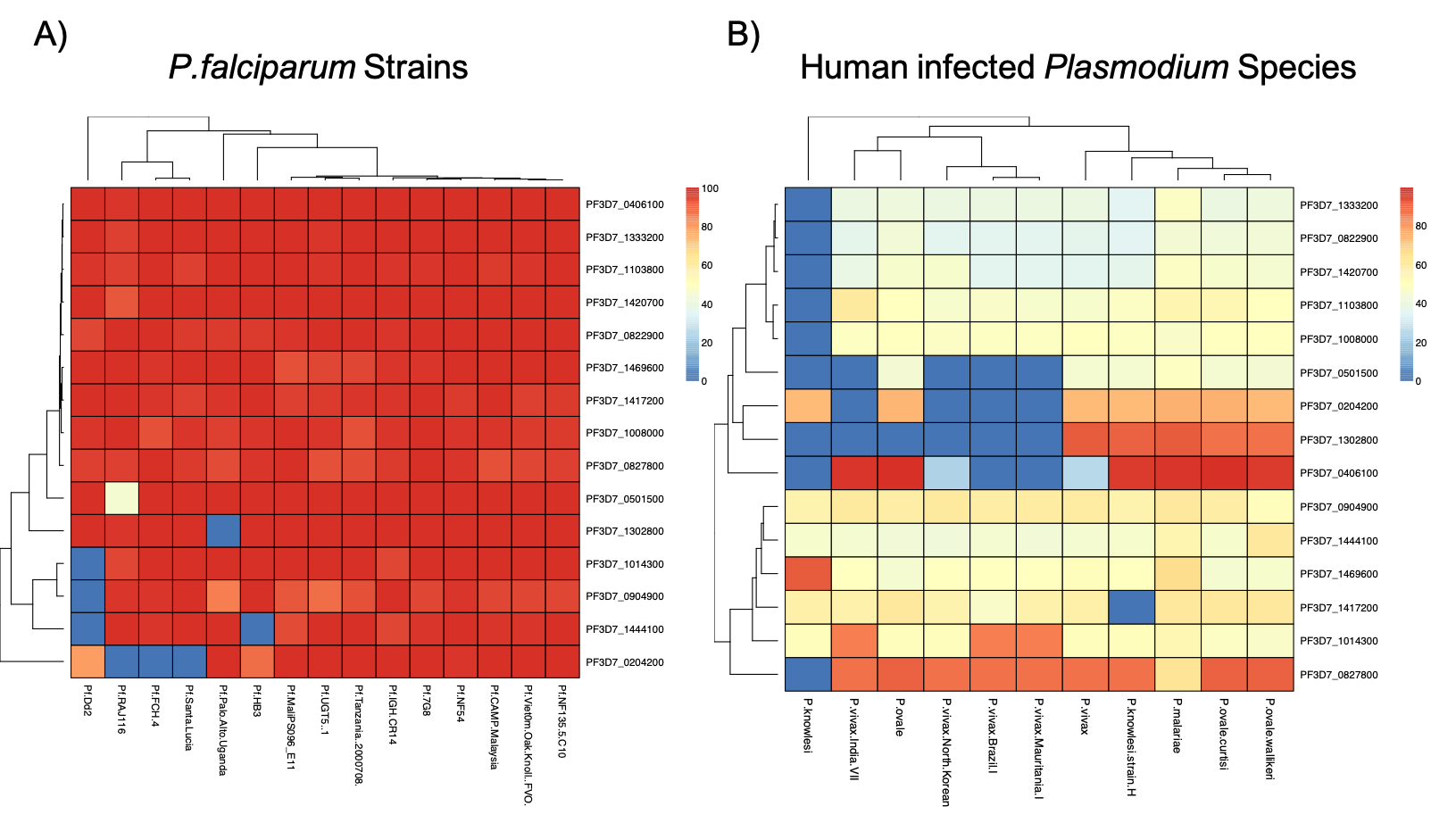
**

**SUPPLEMENTARY TABLES**

**Table S1**: Characteristic of the study population. Eighty children were enrolled in a longitudinal study in Ghana; 39 young children aged 1 to 2 years and 41 older children aged 4 to 5 years. Over the year, study participant were visited 7 times (every 2 months) and clinical, haematological and parasitological data collected at the beginning of the study and during each visit (C0 to C6). Asexual parasites were counted against 200 white blood cells and converted to parasites/μL assuming a density of 8,000 white blood cells/μL blood. Fever was defined as a temperature above 37.5 C°. Anemia was defined as hemoglobin level < 9 g/dL. Comparisons of clinical parameters between age group (younger /older) and between seasons (wet/dry) were made using the Wilcoxon rank-sum test and Wilcoxon paired rank-test, respectively. Wet season it's from contact 0 (May) to contact 2 (September) and dry season from contact 3 (November) to contact 5 (March).

**Table S2: Association between individual protein and protection from malaria**. Multivariate logistic regression models, adjusted for age and parasite density at the baseline were fit to estimate the association between individual protein-specific antibody responses signal intensity (SI) for the 1,080 protein and protection from symptomatic malaria. In these models, the SI was the dependent and the immune status (resistant/susceptible), age, and parasite density were independent variables. None of the individual protein was significantly associate with protection after multiple correction (p>0.05 Bonferroni correction). Also reported are the Area Under the Curve and the mean intensity for resistant and susceptible children and the transcript characteristic dowloaded from PlasmoDB. Data are ordered by p value from the lowest to the highest.

**Table S3**: PLS-DA identified 15 antigens signature discriminating susceptible and resistant children. Children of 1-5 years of age (n = 72) were defined “resistant” (n = 56) if they did not experience a clinical malaria episode during the 12 -month study period and “susceptible” (n = 16 children) otherwise. Antibody response was measured at the beginning of the wet season (contact 0). Five antigens were associated to the susceptible group and 10 antigens were associated to the resistant group. A VIP score, which is a measure of a variable’s importance in the PLS-DA model, is reported. The VIP score of a variable is calculated as a weighted sum of the squared correlations between the PLS-DA components and the original variable. The weights correspond to the percentage variation explained by the PLS-DA component in the model. The number of terms in the sum depends on the number of PLS-DA components found to be significant in distinguishing the classes. Also reported are the mean intensity for resistant and susceptible children. Data are ordered by importance (pVIP score) value from the highest to the lowest.
